# Integrated glycomics and genetics analyses reveal a potential role for N-glycosylation of plasma proteins and IgGs, as well as the complement system, in the development of type 1 diabetes

**DOI:** 10.1007/s00125-023-05881-z

**Published:** 2023-03-13

**Authors:** Najda Rudman, Simranjeet Kaur, Vesna Simunović, Domagoj Kifer, Dinko Šoić, Toma Keser, Tamara Štambuk, Lucija Klarić, Flemming Pociot, Grant Morahan, Olga Gornik

**Affiliations:** 1 Faculty of Pharmacy and Biochemistry, University of Zagreb, Zagreb, Croatia; 2 Steno Diabetes Center Copenhagen, Herlev, Denmark; 3 Institute of Genetics and Cancer, MRC Human Genetics Unit, University of Edinburgh, Edinburgh, UK; 4 Faculty of Health and Medical Sciences, University of Copenhagen, Copenhagen, Denmark; 5 Centre for Diabetes Research, The Harry Perkins Institute for Medical Research, University of Western Australia, Perth, WA Australia; 6 Australian Centre for Accelerating Diabetes Innovations, University of Melbourne, Melbourne, VIC Australia

**Keywords:** *C3*, GWAS, IgG N-glycosylation, *MGAT3*, plasma protein N-glycosylation, *ST6GAL1*, type 1 diabetes

## Abstract

**Aims/hypothesis:**

We previously demonstrated that N-glycosylation of plasma proteins and IgGs is different in children with recent-onset type 1 diabetes compared with their healthy siblings. To search for genetic variants contributing to these changes, we undertook a genetic association study of the plasma protein and IgG N-glycome in type 1 diabetes.

**Methods:**

A total of 1105 recent-onset type 1 diabetes patients from the Danish Registry of Childhood and Adolescent Diabetes were genotyped at 183,546 genetic markers, testing these for genetic association with variable levels of 24 IgG and 39 plasma protein N-glycan traits. In the follow-up study, significant associations were validated in 455 samples.

**Results:**

This study confirmed previously known plasma protein and/or IgG N-glycosylation loci (candidate genes *MGAT3*, *MGAT5* and *ST6GAL1*, encoding beta-1,4-mannosyl-glycoprotein 4-beta-*N*-acetylglucosaminyltransferase, alpha-1,6-mannosylglycoprotein 6-beta-*N*-acetylglucosaminyltransferase and ST6 beta-galactoside alpha-2,6-sialyltransferase 1 gene, respectively) and identified novel associations that were not previously reported for the general European population. First, novel genetic associations of IgG-bound glycans were found with SNPs on chromosome 22 residing in two genomic intervals close to candidate gene *MGAT3*; these include core fucosylated digalactosylated disialylated IgG N-glycan with bisecting *N*-acetylglucosamine (GlcNAc) (*p*_discovery_=7.65 × 10^−12^, *p*_replication_=8.33 × 10^−6^ for the top associated SNP rs5757680) and core fucosylated digalactosylated glycan with bisecting GlcNAc (*p*_discovery_=2.88 × 10^−10^, *p*_replication_=3.03 × 10^−3^ for the top associated SNP rs137702). The most significant genetic associations of IgG-bound glycans were those with *MGAT3*. Second, two SNPs in high linkage disequilibrium (missense rs1047286 and synonymous rs2230203) located on chromosome 19 within the protein coding region of the complement C3 gene (*C3*) showed association with the oligomannose plasma protein N-glycan (*p*_discovery_=2.43 × 10^−11^, *p*_replication_=8.66 × 10^−4^ for the top associated SNP rs1047286).

**Conclusions/interpretation:**

This study identified novel genetic associations driving the distinct N-glycosylation of plasma proteins and IgGs identified previously at type 1 diabetes onset. Our results highlight the importance of further exploring the potential role of N-glycosylation and its influence on complement activation and type 1 diabetes susceptibility.

**Graphical abstract:**

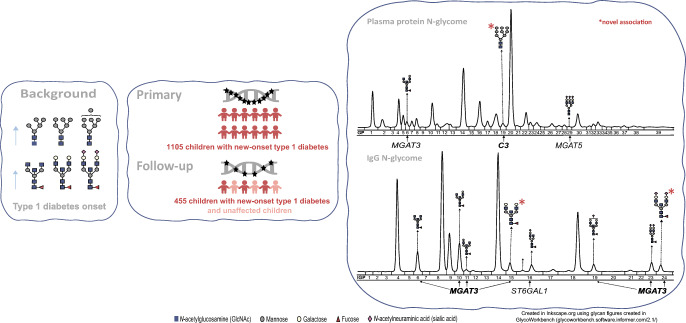

**Supplementary Information:**

The online version contains peer-reviewed but unedited supplementary material available at 10.1007/s00125-023-05881-z.

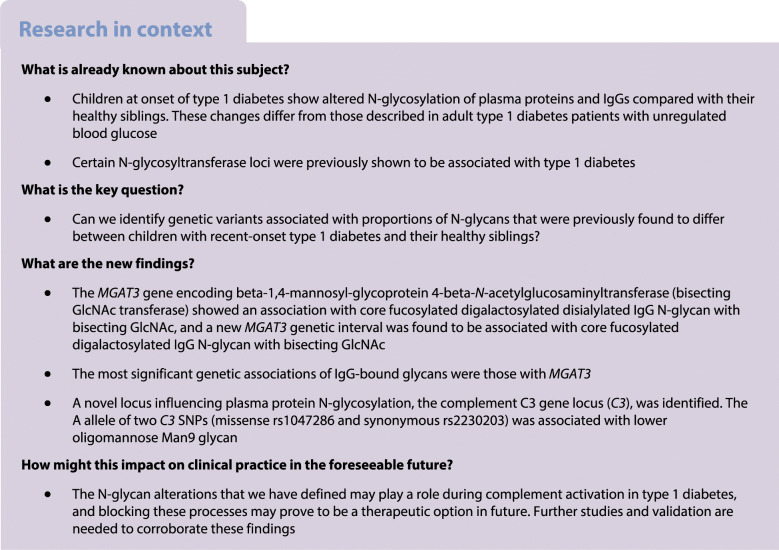



## Introduction

Type 1 diabetes is a chronic disease that is characterised by the autoimmune destruction of insulin-producing pancreatic beta cells [1]. The number of children and adolescents diagnosed with type 1 diabetes has been increasing worldwide at an annual rate of about 3% [2]. Despite the identification of many genetic risk factors [3], the underlying causes of this disease remain unclear, and accumulating evidence suggests that environmental factors play an important role in the development of type 1 diabetes [4].

N-glycosylation is a diverse protein modification process by which complex oligosaccharide structures (glycans) are added to the protein backbone [5]. It is important to stress that glycosylation should not be confused with glycation, since glycosylation is a complex enzymatic process strictly regulated by a network of glycosyltransferases, glycosidases, transcriptional factors, sugar nucleotides and other molecules [6]. Glycation, on the other hand, is a non-enzymatic reaction between reducing sugar and protein, such as the one described for glycated haemoglobin [7]. N-glycosylation changes can influence protein function. For example, addition of sialic acid to the terminal end of N-glycan changes the function of antibodies of the IgG isotype from pro- to anti-inflammatory agents [8], whereas addition of bisecting *N*‑acetylglucosamine (GlcNAc) is associated with an increased ability of IgGs to destroy target cells through antibody-dependent cellular cytotoxicity [9]. Both the human plasma protein and IgG N-glycomes demonstrate remarkably low intra-individual variance under physiological conditions [10, 11], and at the same time are extremely sensitive to different pathological processes [12], thus supporting their diagnostic and prognostic potential. N-glycosylation changes have been described in various diseases, including type 1 diabetes and other diabetes types [12]. It has been shown that it is possible to distinguish HNF1A-maturity onset diabetes of the young from healthy controls and even from other diabetes types based on proportions of antennary fucose of plasma proteins, and recently that N-glycans with best diagnostic value mostly originate from alpha-1-acid glycoprotein [12, 13]. Remarkably, recent studies have even demonstrated that identification of individuals at an increased risk of future diabetes development is possible based on their N-glycan profiles [12].

Much evidence has been gathered regarding the role of highly branched N-glycans in autoimmunity in general, as well as in type 1 diabetes specifically [12]. Highly branched glycans present on proteins on cell surfaces are involved in interaction with galectins, and thus the formation of glycan–galectin lattices, resulting in increased protein retention time on the cell surface [14]. Defective N-glycosylation of T cells has been implicated in the pathogenesis of type 1 diabetes [14]. N-glycan branching increased the surface retention time of the T cell activation inhibitory glycoprotein CTLA-4 [15] encoded by the *CTLA4* gene, which has been identified as one of the causal candidate genes in type 1 diabetes [16].

Genome-wide association studies (GWASs) of the plasma protein and IgG N-glycomes have identified glycosyltransferase loci, as well as loci containing genes that have not previously been shown to be associated with protein glycosylation [17, 18]. Some of these genes (for example *IKZF1* and *BACH2*) have also been shown to be associated with various diseases, including type 1 diabetes [19]. Genetic studies identified a glycosyltransferase gene, *FUT2*, as one of the causal candidate genes in type 1 diabetes [20], with the possible mechanism involving host resistance to infections [21]. The glycosyltransferase loci *MGAT5* (encoding alpha-1,6-mannosylglycoprotein 6-beta-*N*-acetylglucosaminyltransferase) and *MGAT1* have been implicated in the pathogenesis of type 1 diabetes through N-glycan branching and its impact on T cell activation [14, 22]. Decreased expression of *ST6GAL1* (encoding ST6 beta-galactoside alpha-2,6-sialyltransferase 1) in B cells has been shown to be associated with type 1 diabetes risk-associated alleles [23].

Protein glycosylation is a complex process that is regulated by a vast network of genes [6], many of which have still not been identified in humans, although they have been identified in a comprehensive study of mouse glycans [24]. We recently showed that plasma protein and IgG N-glycosylation differs between children with recent-onset type 1 diabetes and their healthy siblings [25], and is different from the N-glycan profile of adult type 1 diabetes patients with unregulated blood glucose [26]. In this study, we aimed to obtain knowledge of the genetic impact on the distinct plasma protein and IgG N-glycosylation that has been shown to accompany onset of type 1 diabetes [25], and identify type 1 diabetes risk-associated genes that regulate N-glycosylation. As far as we are aware, this is the first study to correlate genetic and N-glycome data in type 1 diabetes patients.

## Methods

### Ethics statement

The study was approved by Danish Ethical Committee KA-95139 m and the ethics committee of the University of Zagreb, Faculty of Pharmacy and Biochemistry. The study was performed in accordance with the Declaration of Helsinki. Informed consent was given by all the patients, their parents or guardians.

### Study participants

The discovery study comprised 1105 children with new-onset type 1 diabetes (median age 10 years, range 1–18 years) whose plasma samples were collected within three months of type 1 diabetes diagnosis through the Danish Registry of Childhood and Adolescent Diabetes (DanDiabKids) [27]. The follow-up (validation) study comprised 190 children with recent-onset type 1 diabetes and 265 unaffected children from the same family-based DanDiabKids registry. Three of the childhood type 1 diabetes patients have one or more siblings within the control group. Details of the participants in this study are summarised in Table 1.

**Table 1:** Description of the research population in the discovery and follow-up studies

Characteristic	Discovery study	Follow-up study
Genotyping assay	Infinium Immunochip (Illumina)	KASP (LGC Genomics)
Participant types	Children with new-onset type 1 diabetes from family-based DanDiabKids registry	Children with new-onset type 1 diabetes/unaffected children from family-based DanDiabKids registry
Number of participants	1105	190/265
Number of participants with quantified plasma protein N-glycans	1074	175/264
Number of participants with quantified IgG N-glycans	1086	177/260
Median age (range), years	10 (1–18)	10 (1–19)/11 (2–23)
Percentage female	47.6	43.6/47.7

The inclusion criterion for unaffected children was that a sample from their biological sibling with type 1 diabetes was available in the registry. More than 95% of the sibling samples were collected at the same date as the proband sample, and the sampling dates are quite equally distributed over the year. The year of sampling for unaffected children ranged from 1997 to 2000, and the last registry data extraction and disease status check for unaffected children was performed in January 2019. At the last disease status check, it was established that two individuals had developed type 1 diabetes, one within nearly 6 years and other within 9 years. Some of the unaffected siblings were lost to follow-up if they were subsequently diagnosed at more than 18 years old, at which age they are often referred to adult type 1 diabetes clinics, and therefore their type 1 diabetes status is less certain than for those individuals who were followed for an extended time. However, as the incidence of type 1 diabetes after puberty decreases markedly with increasing age, it is less likely that the older individuals followed for a shorter period developed the disease [28]. Subsets of the cohorts collected through this registry have been used in a number of studies [29–31].

### Discovery study

In the discovery study, 1105 samples from children with recent-onset type 1 diabetes were genotyped for 183,546 SNPs using Immunochip, a custom-made Infinium array (Illumina, USA), as described previously [32]. A total of 177,022 markers passed the initial sample quality control process, including sample call rate and a concordance check of reported sex vs genotyped sex. Additional quality control was performed by removing SNPs with a genotyping call rate <95% (5% missing) and a minor allele frequency <5%. In total, 108,428 SNPs passed the filtering criteria and were retained in the analysis. The mean genotyping rate in the participants was 99%. All the quality filtering steps were performed using PLINK version 1.07 [33]. To avoid missing true association signals, the SNPs were not filtered for deviations from the Hardy–Weinberg equilibrium, because disease association and population structure can cause deviations from the Hardy–Weinberg equilibrium [34]. The genotype-calling algorithms exported the allele calls aligned to the TOP strand.

After genotype quality control, data were analysed for associations between glycan proportions and individual SNPs genome-wide using the ‘qassoc’ function in PLINK [33], with a *p* value cut-off of 5 × 10^−8^. Genome-wide significance thresholds were further adjusted for 21 independent IgG glycan traits [19] (*p*≤2.4 × 10^−9^) and 39 plasma protein glycan traits (*p*≤1.3 × 10^−9^). Information on linkage disequilibrium (LD) was obtained using SNiPA [35] (SNiPA version 3.4 from November 2020, GRCh37.p13, Ensembl version 87, 1000 genomes phase 3, version 5, European).

### Follow-up study

In the follow-up study used for validation, 21 SNPs revealed in the discovery phase were used for genotyping (see electronic supplementary material [ESM] Table 1). Samples from 455 individuals were genotyped using Kompetitive allele-specific PCR genotyping (KASP, LGC Genomics, UK). Of the 21 SNPs, 18 were successfully genotyped (rs137707, rs1047286 and rs137702 failed validation and were not analysed).

The genotype effect on glycan abundance was estimated by mixed modelling, with glycan abundance as the dependent variable, and genotype, disease status and interaction between disease status and genotype as independent variables. Sex and age were included as independent fixed variables, and family identifier was included as a random intercept [36]. The number of independent novel glycan–SNP combinations tested was used to adjust the significance threshold (0.05 for plasma protein glycans and 0.05/2 for IgG glycans).

### N-glycome analysis

Briefly, a 10 μl aliquot of plasma was used for plasma protein N-glycome profiling, and 70 μl plasma was used to isolate IgG using a protein G monolithic plate (CIM Protein G 96-monolithic plate, BIA Separations, Slovenia) [37]. N-glycans were then enzymatically released and fluorescently labelled [38]. Hydrophilic interaction ultra-performance liquid chromatography was used to separate N-glycans [37]. Automated integration was applied to separate the chromatograms into 24 peaks for IgG N-glycans (IGP1–IGP24) and 39 peaks for plasma protein N-glycans (GP1–GP39) [39], and all these glycan traits were included in the genetic association analyses. The amount of glycan in each peak was expressed as a percentage of the total integrated area.

N-glycome data for these participants had been obtained previously [25], using 24 and five plates (batches) in the first and second parts of the study, respectively. The first part of the study included N-glycosylation analyses of 1917 children with new-onset type 1 diabetes. The second part of the study included 188 of the 1917 participants involved in the primary study and their 244 unaffected siblings. Within each study part, samples were randomised by age, sex and disease status, and standard and duplicated samples were added to each plate to minimise experimental error. CVs of the measured N-glycans among standard and duplicated samples are presented in ESM Table 2. In order to combine these two parts of the previous study, batch effects were removed using the ComBat method in the R package sva [40]. Data gathered from the samples included in both parts of the study were used for estimation of differences between the study parts. The effect was estimated by mixed modelling using the R package lme4 [36], in which logit-transformed glycan abundance was the dependent variable, while study part was a fixed factor and sample identifier was modelled as a random intercept. The estimated effect, known to originate for technical reasons, was removed from the data.

## Results

**Fig. 1:**
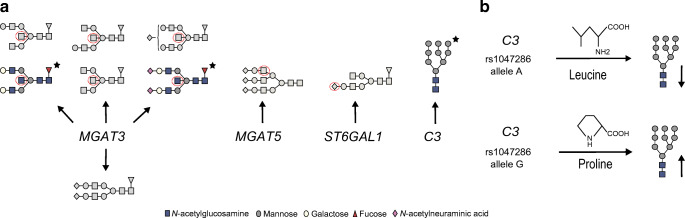
(**a**) Overview of significant genetic associations with plasma protein and IgG N-glycan proportions in individuals with recent-onset type 1 diabetes. Genes are shown grouped with their associated glycans. Glycans are shown as per GlycoWorkBench standard figures [59]. Glycans that showed novel genetic associations are indicated by a star and presented in colour. The red dotted circles indicate glycan residues that were synthesised by the action of the enzymes encoded by the associated genes. (**b**) Effect of the *C3* missense variant rs1047286. This SNP results in a cyclic to acyclic amino acid substitution, and is associated with altered proportions of the oligomannose plasma protein glycan

A genetic association study of the plasma protein and IgG N-glycome was performed using data from 1105 recent-onset type 1 diabetes childhood patients from the family-based DanDiabKids registry, who were genotyped at more than 183,000 genetic variants. Data for plasma protein N-glycans as well as those specifically on IgG (which are also represented within plasma protein N-glycans) were used herein [25]. The results are presented in Tables 2 and 3. We identified five genome-wide significant loci associated with plasma protein and/or IgG N-glycans; candidate genes include *MGAT3* (encoding beta-1,4-mannosyl-glycoprotein 4-beta-*N*-acetylglucosaminyltransferase), *MGAT5*, *ST6GAL1* and *C3* (encoding complement C3) (Fig. 1). Regional association plots are presented in ESM Figures 1–11. The pleiotropy of identified variants in terms of gene expression, protein expression and diseases is summarised in Table 4.

**Table 2:** Genetic markers that showed significant genome-wide association with plasma protein N-glycans in the discovery cohort and the follow-up study comprising children with recent-onset type 1 diabetes and unaffected children from the family-based DanDiabKids registry

Glycan/Gene					Discovery cohort (*N*=1105)					Follow-up study (*N*=455)		General European population [18] (*N*=4802)
Glycan	Glycan structure	Locus	Candidate gene	SNP	*N*	OA, MA (MAF)	*R*^2^	*p*	β (SE)	*p*	β (SE)	*p*
GP29	A3G3S3	2: 135,014,116	*MGAT5*	rs2460382	1044	A, G (0.21)	0.04	3.14 × 10^−11^	0.01 (0.002)	2.27 × 10^−2^	0.01 (0.003)	^a^5.95 × 10^−17^
GP19	Man9	19: 6,713,262	*C3*	rs1047286	1033	G, A (0.20)	0.04	2.43 × 10^−11^	−0.03 (0.005)	^b^8.66 × 10^−4^	−0.04 (0.012)	Association not reported^c^
GP19	Man9	19: 6,710,782	*C3*	rs2230203	1045	C, A (0.18)	0.04	4.15 × 10^−11^	−0.03 (0.005)	8.66 × 10^−4^	−0.04 (0.012)	Association not reported^c^
GP6	FA2[6]BG1	22: 39,843,409	*MGAT3*	rs5757678	1045	A, G (0.27)	0.04	1.48 × 10^−11^	0.08 (0.012)	8.63 × 10^−7^	0.11 (0.021)	^d^7.98 × 10^−10^
GP6	FA2[6]BG1	22: 39,844,793	*MGAT3*	rs5757680	1034	G, A (0.26)	0.04	2.21 × 10^−11^	0.08 (0.012)	1.35 × 10^−6^	0.10 (0.021)	^d^7.98 × 10^−10^

Significant associations from the largest GWAS on plasma protein N-glycans comprising 4802 participants from the general European population [18] were searched for the same glycan–SNP association, or SNPs in LD (*R*^2^>0.5) with the SNP identified in this study, and the same direction of effect estimates (either increasing or decreasing with the same allele), and their *p* value is included in this table. Associations identified in this study and not previously reported in the general European population are presented in this table with ‘Association not reported’. Locus information is presented as ‘chromosome number: locus start’. The results are reported for GRCh Build 37, and alleles are aligned to the TOP strand. The β coefficient is reported for the minor allele and expressed as the relative glycan abundance (%)

^a^Results are reported for rs1257220 (*R*^2^=0.98 with rs2460382)

^b^Results are reported for rs2230203 (*R*^2^=0.85 with rs1047286)

^c^In the general European population, GP19 was exclusively associated with the chromosome 6 SNP rs3115663 near the *PRRC2A* gene located in the HLA class III region

^d^Results are reported for rs909674 (*R*^2^=0.91 with rs5757678, *R*^2^=0.91 with rs5757680)

*N*, sample size; OA, other allele; MA, minor allele; MAF, minor allele frequency; *R*^2^, percentage of explained glycan variance; F, core fucose α1,6-linked to the inner GlcNAc; Man*x*, number (*x*) of mannose residues on core GlcNAcs; A*x*, number of antenna (GlcNAc) on the trimannosyl core; A2, biantennary N-glycan; A3, triantennary N-glycan; A2[6], galactose linked on the antenna of the α1,6 mannose; B, bisecting GlcNAc β1,4-linked to β1,4-mannose; G*x*, number (*x*) of linked galactose residues on antenna; S*x*, number (*x*) of sialic acid residues linked to galactose

**Table 3:** Genetic markers that showed significant genome-wide association with IgG N-glycans in the discovery cohort and follow-up study comprising children with recent-onset type 1 and unaffected children from the family-based DanDiabKids registry

Glycan/Gene						Discovery cohort (*N*=1105)					Follow-up study (*N*=455)		General European population [17] (*N*=8090)
Glycan	Glycan structure	Locus	Candidate gene	SNP	Number of SNPs in the interval	*N*	OA, MA (MAF)	*R*^2^	*p*	β (SE)	*p*	β (SE)	*p*
IGP16	FA2[3]G1S1	3: 186,741,221–186,743,053	*ST6GAL1*	rs3872724	2	1056	G, A (0.37)	0.09	1.81 × 10^−22^	−0.16 (0.016)	2.21 × 10^−5^	−0.14 (0.032)	^a^8.63 × 10^−65^
IGP6	FA2B	22: 39,843,409–39,844,793	*MGAT3*	rs5757678	2	1056	A, G (0.27)	0.05	1.57 × 10^−13^	0.39 (0.052)	4.61 × 10^−7^	0.49 (0.096)	^b^2.35 × 10^−18^
IGP10	FA2[6]BG1	22: 39,778,167–39,844,793	*MGAT3*	rs5757680	4	1045	G, A (0.26)	0.1	2.12 × 10^−26^	0.41 (0.038)	1.60 × 10^−14^	0.56 (0.070)	^b^5.96 × 10^−27^
IGP10	FA2[6]BG1	22: 39,738,501–39,756,985	*MGAT3*	rs137702	12	1048	G, A (0.24)	0.05	5.95 × 10^−14^	0.31 (0.041)	^c^4.07 × 10^−5^	0.33 (0.080)	^d^3.64 × 10^−18^
IGP11	FA2[3]BG1	22: 39,778,167–39,844,793	*MGAT3*	rs5757680	4	1045	G, A (0.26)	0.05	1.63 × 10^−13^	0.04 (0.005)	4.24 × 10^−5^	0.03 (0.008)	^b^6.18 × 10^−14^
IGP15	FA2BG2	22: 39,778,167–39,844,793	*MGAT3*	rs5757680	4	1045	G, A (0.26)	0.06	1.04 × 10^−15^	0.12 (0.015)	8.27 × 10^−10^	0.16 (0.026)	^b^1.89 × 10^−12^
IGP15	FA2BG2	22: 39,739,638–39,756,985	*MGAT3*	rs137702	7	1048	G, A (0.24)	0.04	2.88 × 10^−10^	0.1 (0.016)	^c^3.03 × 10^−3^	0.09 (0.029)	Association not reported^e^
IGP19	FA2BG2S1	22: 39,843,409–39,844,793	*MGAT3*	rs5757680	2	1045	G, A (0.26)	0.04	1.38 × 10^−10^	0.1 (0.016)	8.17 × 10^−5^	0.11 (0.027)	^b^4.63 × 10^−10^
IGP23	FA2G2S2	22: 39,843,409–39,844,793	*MGAT3*	rs5757680	2	1045	G, A (0.26)	0.04	5.04 × 10^−11^	−0.19 (0.028)	3.81 × 10^−3^	−0.15 (0.051)	^b^1.20 × 10^−20^
IGP24	FA2BG2S2	22: 39,843,409–39,844,793	*MGAT3*	rs5757680	2	1045	G, A (0.26)	0.04	7.65 × 10^−12^	0.12 (0.017)	8.33 × 10^−6^	0.13 (0.029)	Association not reported^f^

Significant associations from the largest GWAS on IgG N-glycans comprising 8090 individuals from the general European population [17] were searched for the same glycan–SNP association, or SNPs in LD (*R*^2^>0.5) with the SNP identified in this study, and the same direction of effect estimates (either increasing or decreasing with the same allele), and their *p* value is included in this table. Associations identified in this study and not previously reported in the general European population are presented in this table with ‘Association not reported’. Each locus is represented by the SNP with the strongest association in the region. Locus information is presented as ‘chromosome number: locus start – locus end’. The results are reported for GRCh Build 37, and alleles are aligned to the TOP strand. LD was calculated using SNiPA [35], and SNPs were grouped in the same genomic interval based on LD (*R*^2^>0.5) with the top associated SNP within the interval. The β coefficient is reported for the minor allele and expressed as the relative glycan abundance (%)

^a^Results are reported for rs3821819 (*R*^2^=0.69 with rs3872724)

^b^Results are reported for rs8137426 (*R*^2^=0.99 with rs5757678, *R*^2^=1 with rs5757680)

^c^Results are reported for rs4821887 (*R*^2^=0.98 with rs137702)

^d^Results are reported for rs6001585 (*R*^2^=0.58 with rs137702)

^e^The closest SNP defined by LD for this association in the general European population is rs4821897 (*R*^2^=0.37 with rs137702, *p*=1.15 × 10^−12^)

^f^In the general European population, IGP24 was exclusively associated with chromosome 3 *ST6GAL1* SNPs

*N*, sample size; OA, other allele; MA, minor allele; MAF, minor allele frequency; *R*^2^, percentage of explained glycan variance; F, core fucose α1,6-linked to the inner GlcNAc; A*x*, number of antenna (GlcNAc) on the trimannosyl core; A2, biantennary N-glycan; A2[3] or A2[6], galactose residues linked on the antenna of the α1,3 or α1,6 mannose, respectively; B, bisecting GlcNAc β1,4-linked to β1,4-mannose; G*x*, number (*x*) of linked galactose residues on antenna; S*x*, number (*x*) of sialic acid residues linked to galactose

**Table 4:** Pleiotropy in terms of gene expression in relevant tissues, protein expression, diseases and effect on transcript for variants that showed novel associations with plasma protein and IgG N-glycosylation; the most biologically plausible candidates for observed novel associations are the *C3* gene due to an associated missense variant located in the coding region and the associated Man9 glycan attached to its protein product surface, and the N-glycosyltransferase gene *MGAT3*

Glycan	Glycan structure	Locus	Gene	SNP	Number of SNPs in the interval	Effect on transcript	eQTL in relevant tissues/tissue	pQTL; protein; tissue	Disease gene (s); disease
GP19	Man9	19: 6,710,782–6,713,262	*C3*	rs1047286	2	Direct: rs1047286 missense coding variant, C3 protein pPro314Leu substitution Putative: *C3* rs2230203 synonymous coding variant		*C8*; C8; plasma	*C3*, *C8A/B/G*; increased risk of type 1 diabetes among HLA-DR4/4 carriers [41]^a^, macular degeneration, complement component 3 deficiency, haemolytic uraemic syndrome, complement component 8 deficiency type I/II
IGP24	FA2BG2S2	22: 39,843,409–39,844,793		rs5757680	2	Intergenic region	*MGAT3/*whole blood	*IL6ST* (*sGP130*); IL-6 receptor subunit β (gp130, soluble); plasma	*IL6ST*; hyper-IgE recurrent infection syndrome 4b
IGP15	FA2BG2	22: 39,739,638–39,756,985	*SYNGR1*	rs137702	7	Putative: *SYNGR1* intron variants (rs137702, rs137707, rs2413589, rs4821887)	*MGAT3/*B cells	*IL6ST (sGP130*); IL-6 receptor subunit β (gp130, soluble); plasma	*IL6ST*; hyper-IgE recurrent infection syndrome 4b

Each locus is represented by the SNP with the strongest association in the region. Locus information is presented as ‘chromosome number: locus start – locus end’. The results are reported for GRCh Build 37. eQTL, pQTL, disease gene/disease, effect on transcript and LD were obtained using SNiPA [35], and SNPs were grouped in the same genomic interval based on LD (*R*^2^>0.5) with the top associated SNP within the interval

^a^rs2230199 was previously associated with an increased risk of type 1 diabetes among HLA-DR4/4 carriers (*R*^2^=0.82 with rs1047286; *R*^2^=0.7 with rs2230203)

*C8A/B/G*, complement C8 α/β/γ chain gene; *IL6ST*, interleukin 6 cytokine family signal transducer gene; gp130, soluble glycoprotein 130 (synonyms: IL6ST, soluble)

### Genetic association analysis of plasma protein N-glycans identified a novel locus

Genetic association analyses identified a novel N-glycosylation locus, the *C3* gene. Two SNPs in high LD (missense rs1047286 and synonymous rs2230203) located on chromosome 19 within the protein coding region of the *C3* gene showed significant genome-wide association with the oligomannose Man9 glycan (GP19) and explained 4% of the variance of the associated glycan. The A alleles of these two SNPs were associated with lower Man9 levels.

This study also confirmed previously reported associations for *MGAT3* and *MGAT5* [18]. GP6 variation (core fucosylated monogalactosylated glycan with bisecting GlcNAc) was associated with two SNPs located in the intergenic region on chromosome 22 (candidate gene *MGAT3*): rs5757678 and rs5757680. Also, rs2460382 within an intron of *MGAT5* on chromosome 2 showed significant genome-wide association with triantennary trigalactosylated trisialylated plasma protein N-glycan (GP29) (*p*=3.14 × 10^−11^).

### Novel IgG N-glycan associations uncovered for the N-glycosyltransferase *MGAT3*

Novel genetic associations of IgG-bound glycans were found with SNPs on chromosome 22; these reside in two genomic intervals defined by their LD (*R*^2^>0.5) with the top associated SNP within each interval. Associations within the first genomic interval were the strongest. The complete list of genetic markers that showed significant genome-wide association with IgG N-glycans in the discovery cohort is presented in ESM Table 3.

The most significant novel IgG glycan association was between IGP24 (percentage of core fucosylated biantennary digalactosylated disialylated glycan with bisecting GlcNAc) and two SNPs located in the intergenic region within the first associated genomic interval (candidate gene *MGAT3*). These SNPs explained 4% of the variance of the associated IGP24. In a previous GWAS on the general European population, IGP24 was exclusively associated with *ST6GAL1* on chromosome 3 [17]. In the present study, the most significant genetic associations of IgG-bound glycans were with *MGAT3*, whereas the most significant genetic associations in the general European population were for *ST6GAL1* [17].

Another novel association was found between IGP15 (percentage of core fucosylated biantennary digalactosylated glycan with bisecting GlcNAc) and SNPs within the second genomic interval. The most significantly associated SNP was rs137702 (*p*=2.88 × 10^−10^), which resides within an intron of the synaptogyrin 1 gene (*SYNGR1*, a candidate gene bisecting GlcNAc transferase *MGAT3*), and explained 4% of the variance of the associated IGP15. IGP15 was associated with *MGAT3* in the general European population, but not with this particular genetic interval [17].

This study also confirmed other previously reported IgG N-glycan associations for *MGAT3* and *ST6GAL1* [17]. In summary, SNPs within the candidate *MGAT3* gene were associated with core fucosylated glycans with bisecting GlcNAc in one direction, and core fucosylated digalactosylated disialylated glycan without bisecting GlcNAc in the opposite direction. The most significantly associated SNP was rs5757680 (*p*=2.12 × 10^−26^). Two SNPs within an intron of *ST6GAL1* showed significant genome-wide association with IGP16, corresponding to the IgG-attached monosialylated N-glycan. The most significant SNP in this region was rs3872724 (*p*=1.81 × 10^−22^).

## Discussion

This study analysed the genetic impact on distinct plasma protein and IgG N-glycomes accompanying type 1 diabetes onset that we described previously [25]. Within a cohort of 1105 recent-onset type 1 diabetes patients, associations that were not previously reported for the general European population were found. The N-glycosyltransferase gene *MGAT3* showed novel association with core fucosylated biantennary digalactosylated disialylated IgG N-glycan with bisecting GlcNAc (IGP24), which was previously exclusively associated with sialyltransferase *ST6GAL1* [17], and the new *MGAT3* genetic interval was associated with core fucosylated digalactosylated IgG N-glycan with bisecting GlcNAc (IGP15). *MGAT3* showed the strongest IgG N-glycan association, which was reported for *ST6GAL1* in the general European population [17]. A novel locus influencing plasma protein N-glycosylation was also identified, the *C3* gene on chromosome 19. Other previously known associations with plasma protein and IgG N-glycosylation were corroborated (candidate genes *MGAT3*, *MGAT5* and *ST6GAL1*) [17, 18]. These novel genetic associations were replicated in the follow-up cohort.

*C3* encodes the complement component C3, a pivotal protein of all three complement activation pathways that are responsible for host defence against micro-organisms and clearance of self and non-self targets, among other important immune functions [42]. The A alleles of two SNPs in high LD with each other (*R*^2^=0.85) within the exons of the *C3* were associated with lower proportions of oligomannose Man9 glycan of plasma proteins. These SNPs were a non-synonymous SNP (rs1047286) causing a pPro314Leu substitution and a synonymous SNP (rs2230203). Using the publicly available dataset from GWAS of plasma protein N-glycans in the general European population [43, 44] we found that the same Man9 glycan was associated with rs2230203 (*p*=1.33 × 10^−3^), but did not reach genome-wide significance in that cohort, which may mean that it has a bigger effect in type 1 diabetes. Another *C3* SNP, rs2230199, was previously shown to be associated with an increased risk of type 1 diabetes among HLA-DR4/4 carriers, one of the highest risk genotypes associated with type 1 diabetes [41,45]. This SNP is in high LD with both *C3* SNPs identified in this study (*R*^2^=0.82 with rs1047286; *R*^2^=0.7 with rs2230203), supporting the significance of our finding.

The association between *C3* and plasma protein Man9 may be specifically due to the C3 protein among other plasma proteins, as the Man9 glycan is attached on the C3 surface [46]. The A allele of rs1047286 causes a cyclic to acyclic amino acid substitution within C3 that may increase the accessibility for enzymatic processing of Man9, decreasing its levels. As the Man9 glycan is attached to the domain of C3 that is involved in pathogen binding [47], the alterations may be important for complement activation among carriers of the rs1047286 and rs2230203 A alleles. It has been shown previously that activity of the complement activation alternative pathway was higher among individuals with the rs1047286 A allele [48].

IgG N-glycans with bisecting GlcNAc (IGP24 and IGP15) showed novel associations with SNPs on chromosome 22 located in the intergenic regions or in the introns of the *SYNGR1* gene, and close to the N-glycosyltransferase gene *MGAT3*. As *MGAT3* encodes an enzyme that is responsible for addition of bisecting GlcNAc [49], it is the most biologically plausible candidate for these associations. IGP24 was previously shown to be exclusively associated with the sialyltransferase gene *ST6GAL1* [17]. *MGAT3* showed the strongest IgG N-glycan association in this study, which was reported for *ST6GAL1* in the general European population [17]. IGP15 has been previously shown to be associated with *MGAT3*, but not with this particular *MGAT3* genetic interval [17]. Interestingly, the minor alleles of the novel implicated SNPs show pleiotropy with increased expression of soluble glycoprotein 130 (IL-6 receptor subunit β) in plasma [50]. Soluble glycoprotein 130 inhibits IL-6 trans-signalling [51], whereas enhanced T cell responses to IL-6 in type 1 diabetes were shown to be associated with early clinical disease [52].

Increased *MGAT3* expression in whole blood and specifically in cell types relevant for IgG biosynthesis (B cells) has been previously shown to be associated with the relevant alleles of SNPs characterised here and associated with increased IGP24 and IGP15 proportions [50, 53]. In our previous intra-family study of these recent-onset type 1 diabetes patients, IgG N-glycans with bisecting GlcNAc were significantly increased in the type 1 diabetes group compared with their healthy siblings, and, of all tested N-glycans, that corresponding to IGP24 differed most significantly between the studied groups [25]. In addition, decreased *ST6GAL1* expression in B cells has been associated with type 1 diabetes risk-associated alleles [23]. The altered expression of *MGAT3* and *ST6GAL1* in tissues relevant for IgG biosynthesis may be the explanation for the observed associations. IgGs with bisecting GlcNAc are involved in increased antibody-dependent cellular cytotoxicity [9], an important process during elimination of viruses, and it has been suggested that one of the autoimmunity triggers in type 1 diabetes may be virus-derived [54].

The sialyltransferase gene *ST6GAL1* was associated with monosialylated IgG N-glycan, and the same SNP–glycan association has been identified previously [17]. In our previous family-based study [25], there was no significant difference in the proportion of *ST6GAL1*-associated IgG glycan between children with type 1 diabetes and their unaffected siblings. However, proportions of disialylated IgGs increased, whereas those of asialylated IgGs decreased, in the participants with recent-onset type 1 diabetes. The increase in disialylated IgGs was mainly driven by IGP24, which was shown here to be regulated by *MGAT3* instead of *ST6GAL1*.

The same sialyltransferase *ST6GAL1* SNPs regulated FA2BG2 (IGP15) and FA2BG2S2 (IGP24) glycan proportions in opposite directions within the general European population [17]. FA2BG2 is a core fucosylated digalactosylated glycan with bisecting GlcNAc, and is considered as a substrate for a subsequent addition of sialic acids (thus the synthesis of core fucosylated digalactosylated disialylated glycan with bisecting GlcNAc/FA2BG2S2) in the current standard glycosylation pathway, which implies different subcellular localisation of glycosyltransferases and thus specific order of glycan reactions [55]. Recent in silico and in vitro experiments contradicted previous knowledge and showed that certain glycosyltransferases co-localise across the Golgi and that certain reactions outside the standard pathway may occur [56]. However, disialylated glycans were not measured in these experiments, and thus these reactions could not be predicted [56]. Within the type 1 diabetes population of the present study, the same bisecting GlcNAc-transferase *MGAT3* SNPs influenced FA2G2S2 (IGP23; core fucosylated digalactosylated disialylated glycan) and FA2BG2S2 (IGP24) glycan proportions in opposite directions, suggesting that bisecting GlcNAc may be added after the addition of sialic acid.

It has been demonstrated that hyposialylated IgGs activate the endothelial IgG receptor Fcγ receptor IIB (FcγRIIB), resulting in insulin resistance, whereas restored sialylation of IgGs maintained insulin sensitivity [57]. Also, once sialylated, IgG antibodies exert anti-inflammatory properties [8]. The role of these changes in the pathogenesis of type 1 diabetes should be further explored.

There is much evidence in the literature for the role of *MGAT5* in autoimmunity, and in type 1 diabetes specifically [12]. *MGAT5*, which encodes an enzyme responsible for formation of highly branched glycans, was associated with the triantennary GP29 glycan. In previously characterised cohorts, *MGAT5* was associated with triantennary glycans in addition to tetra-antennary ones [18]. It has been demonstrated that mammalian N-glycan branching protects against innate immune self-attack in autoimmune pathogenesis [14]. Furthermore, the presence of the highly branched N-glycan produced by the enzyme encoded by *MGAT5* restricts T cell activation, and *MGAT5*-deficient mice exhibit several autoimmune phenotypes [14]. Interestingly, in our previous intra-family study [25], levels of the associated GP29 N-glycan were significantly increased in children with recent-onset type 1 diabetes relative to their unaffected siblings.

Plasma samples used in the study were collected within 3 months of disease diagnosis, and we acknowledge the fact that the temporal order of changes in glycans and diabetes development cannot be inferred. Other studies of N-glycosylation changes before development of autoimmunity or clinical disease are needed to elucidate whether the observed changes are associated with type 1 diabetes development. However, our previous study did identify significant N-glycosylation differences between these recent-onset type 1 diabetes patients and their healthy siblings [25], and novel genetic associations with these markers were found in the present study. Additionally, some of the identified variants were in high LD with another variant previously associated with an increased risk of developing type 1 diabetes [41]. We were not able to standardise the glycan data against medication intake as data on the treatment of study participants were not available. Nevertheless, our previous study also demonstrated that insulin has a low effect on a limited number of glycans [58], which do not include those glycans identified herein as novel associations. Also, this study comprises children at the onset of type 1 diabetes, without the comorbidities seen in the adult population, and glycan changes related to type 1 diabetes could therefore be investigated more precisely. A potential drawback of our study is that we did not have access to a replication cohort from another population. However, we tested SNP–glycan associations from the discovery phase on additional samples from the same registry and validated the novel associations. Other study limitations include the small sample size and use of a genotyping chip that does not cover the whole genome, and thus depends on initial GWASs for variant selection. Future larger-scale studies, identification of potentially causal/functional variants at the identified loci, replication and functional studies are needed to corroborate our findings. It should also be noted that the observed changes may be relevant for other autoimmune disorders rather than being specific to type 1 diabetes, and this should also be addressed in future studies.

In summary, this study on recent-onset type 1 diabetes patients identified associations that were not previously reported for the general European population. Novel associations with IgG N-glycans were uncovered for variants located on chromosome 22. These variants are located near the N-glycosyltransferase gene *MGAT3*, show pleiotropy with *MGAT3* expression in whole blood and specifically in cell types relevant for IgG biosynthesis, and their associated IgG glycans with bisecting GlcNAc were significantly different between the recent-onset type 1 diabetes patients and their healthy siblings. This study also identified a novel genetic locus associated with plasma protein N-glycosylation, the *C3* gene locus. *C3* variants identified in this study are located in the coding region, and the associated Man9 glycan is attached on a domain that is involved in pathogen binding of the complement component C3 [47], thus the influence of this alteration on complement activation in type 1 diabetes presents an interesting target for future studies. Additionally, the identified *C3* variants were in high LD with another type 1 diabetes risk-associated variant. These findings suggest the need for further studies of N-glycosylation mechanisms regulating type 1 diabetes susceptibility. We would like to highlight the importance of further exploring gene-specific polymorphisms and their associated N-glycosylation changes, as such study may reveal underlying molecular mechanisms, which are still unknown for many identified type 1 diabetes risk-associated SNPs.

## Supplementary information

ESM (PDF 1.01 MB)

## Data Availability

The datasets generated during and/or analysed during the current study are available from the corresponding author on reasonable request.

## Abbreviations

*C3*: Complement C3 gene
GlcNAc: *N*-acetylglucosamine
GP: Plasma protein glycan peak
GWAS: Genome-wide association study
IGP: IgG glycan peak
LD: Linkage disequilibrium
