# Supplementary material for: Integrated glycomics and genetics analyses reveal a potential role for N-glycosylation of plasma proteins and IgGs, as well as the complement system, in the development of type 1 diabetes
Source: Diabetologia. 2023 Mar 13;66(6):1071–83. doi: 10.1007/s00125-023-05881-z (PMC10163086; doi:10.1007/s00125-023-05881-z)
Supplement: Supplementary file 1 — (PDF 1.01 MB) [file 125_2023_5881_MOESM1_ESM.pdf]

ESM Table 1. Selected SNPs used in the validation cohort for genotyping

Candidate gene	Chr	SNP	OA, MA	BP	Associated glycan
MGAT5	2	rs2460382	A, G	135014116	GP29
ST6GAL1	3	rs3872724	G, A	186741221	IGP16
ST6GAL1	3	rs4686838	A, G	186743053	IGP16
C3	19	rs2230203	C, A	6710782	GP19
C3	19	rs1047286	G, A	6713262	GP19
MGAT3	22	rs137683	G, C	39738501	IGP10
MGAT3	22	rs137686	G, C	39739638	IGP10, IGP15
MGAT3	22	rs137690	G, C	39742825	IGP10, IGP15
MGAT3	22	rs9611156	A, C	39743170	IGP10, IGP15
MGAT3	22	rs137707	G, A	39751401	IGP10, IGP15
MGAT3	22	rs5757630	A, C	39754433	IGP10
MGAT3	22	rs2413588	G, A	39755175	IGP10
MGAT3	22	rs760742	A, C	39755190	IGP10
MGAT3	22	rs2413589	G, A	39755689	IGP10, IGP15
MGAT3	22	rs5757633	G, A	39756270	IGP10
MGAT3	22	rs4821887	A, G	39756985	IGP10, IGP15
MGAT3	22	rs1010169	G, A	39778167	IGP10, IGP11, IGP15
MGAT3	22	rs5757650	G, A	39778419	IGP10, IGP11, IGP15
MGAT3	22	rs5757678	A, G	39843409	GP6, IGP6, IGP10, IGP11, IGP15, IGP19, IGP23, IGP24
MGAT3	22	rs5757680	G, A	39844793	GP6, IGP6, IGP10, IGP11, IGP15, IGP19, IGP23, IGP24
MGAT3	22	rs137702	G, A	39750158	IGP10, IGP15

The results are reported for the GRCh Build 37, and alleles are aligned to the TOP strand.

Chr, chromosome; BP, position of a SNP on a chromosome in base-pairs; MA, minor allele; OA, other allele; *MGAT5*, alpha-1,6-mannosylglycoprotein 6-beta-*N*-acetylglucosaminyltransferase gene; *MGAT3*, beta-1,4-mannosyl-glycoprotein 4-beta-*N*-acetylglucosaminyltransferase gene; *C3*, complement C3 gene; *ST6GAL1*, ST6 beta-galactoside alpha-2,6-sialyltransferase 1 gene

ESM Table 2. CVs (%) of the measured N-glycans among standard and duplicated samples obtained previously (Rudman N, Kifer D, Kaur S et al (2022) Children at onset of type 1 diabetes show altered N-glycosylation of plasma proteins and IgG. *Diabetologia* 65(8):1315–1327)

**Glycome**	**IgG**	**Plasma protein**
GP1	13.90	4.59
GP2	8.98	2.01
GP3	22.60	12.10
GP4	3.48	3.68
GP5	15.66	4.86
GP6	3.08	3.87
GP7	9.51	3.03
GP8	1.45	7.98
GP9	2.59	12.76
GP10	3.62	3.11
GP11	7.12	5.02
GP12	10.15	4.92
GP13	11.24	13.04
GP14	2.78	3.22
GP15	5.62	15.06
GP16	3.11	2.59
GP17	5.89	5.07
GP18	3.08	2.53
GP19	5.43	3.37
GP20	12.61	2.60
GP21	12.00	3.51
GP22	16.46	2.93
GP23	5.09	5.54
GP24	6.36	4.64
GP25		22.82
GP26		4.42

GP27	6.68
GP28	4.57
GP29	18.94
GP30	3.86
GP31	8.69
GP32	5.96
GP33	5.04
GP34	7.05
GP35	9.52
GP36	7.25
GP37	8.60
GP38	6.00
GP39	7.72

ESM Table 3. The complete list of genetic markers that reached significant genome-wide association (p<2.4 x 10^{-9}) with IgG N-glycans in the discovery cohort

Chr	Candidate gene	N	Glycan	SNP	*p*	OA, MA (MAF)	β (SE)	R^2	T	BP
3	*ST6GAL1*	1056	IGP16	rs3872724	1.81 x 10^{-22}	G, A (0.37)	-0.16 (0.016)	0.09	-9.98	186741221
3	*ST6GAL1*	1056	IGP16	rs4686838	1.09 x 10^{-15}	A, G (0.44)	-0.13 (0.016)	0.06	-8.14	186743053
22	*MGAT3*	1056	IGP6	rs5757678	1.57 x 10^{-13}	A, G (0.27)	0.39 (0.052)	0.05	7.48	39843409
22	*MGAT3*	1045	IGP6	rs5757680	2.03 x 10^{-13}	G, A (0.26)	0.39 (0.053)	0.05	7.45	39844793
22	*MGAT3*	1056	IGP10	rs1010169	6.39 x 10^{-21}	G, A (0.34)	0.34 (0.036)	0.08	9.59	39778167
22	*MGAT3*	1056	IGP10	rs137683	6.18 x 10^{-10}	G, C (0.23)	0.26 (0.041)	0.04	6.24	39738501
22	*MGAT3*	1052	IGP10	rs137686	5.30 x 10^{-13}	G, C (0.25)	0.29 (0.04)	0.05	7.31	39739638
22	*MGAT3*	1056	IGP10	rs137690	9.32 x 10^{-13}	G, C (0.25)	0.29 (0.04)	0.05	7.23	39742825
22	*MGAT3*	1048	IGP10	rs137702	5.95 x 10^{-14}	G, A (0.24)	0.31 (0.041)	0.05	7.61	39750158
22	*MGAT3*	1056	IGP10	rs137707	8.76 x 10^{-14}	G, A (0.24)	0.31 (0.041)	0.05	7.56	39751401
22	*MGAT3*	1036	IGP10	rs2413588	1.20 x 10^{-10}	G, A (0.29)	0.25 (0.039)	0.04	6.51	39755175
22	*MGAT3*	1056	IGP10	rs2413589	1.14 x 10^{-13}	G, A (0.24)	0.31 (0.041)	0.05	7.52	39755689
22	*MGAT3*	1056	IGP10	rs4821887	8.76 x 10^{-14}	A, G (0.24)	0.31 (0.041)	0.05	7.56	39756985
22	*MGAT3*	1056	IGP10	rs5757630	7.10 x 10^{-10}	A, C (0.30)	0.24 (0.038)	0.04	6.22	39754433
22	*MGAT3*	1056	IGP10	rs5757633	7.10 x 10^{-10}	G, A (0.30)	0.24 (0.038)	0.04	6.22	39756270
22	*MGAT3*	1056	IGP10	rs5757650	1.14 x 10^{-20}	G, A (0.35)	0.34 (0.036)	0.08	9.52	39778419
22	*MGAT3*	1056	IGP10	rs5757678	3.04 x 10^{-26}	A, G (0.27)	0.41 (0.038)	0.1	10.89	39843409
22	*MGAT3*	1045	IGP10	rs5757680	2.12 x 10^{-26}	G, A (0.26)	0.41 (0.038)	0.1	10.93	39844793
22	*MGAT3*	1056	IGP10	rs760742	7.10 x 10^{-10}	A, C (0.30)	0.24 (0.038)	0.04	6.22	39755190
22	*MGAT3*	1056	IGP10	rs9611156	5.09 x 10^{-12}	A, C (0.25)	0.28 (0.04)	0.04	6.98	39743170
22	*MGAT3*	1056	IGP11	rs1010169	2.82 x 10^{-11}	G, A (0.34)	0.03 (0.004)	0.04	6.73	39778167
22	*MGAT3*	1056	IGP11	rs5757650	7.00 x 10^{-11}	G, A (0.35)	0.03 (0.004)	0.04	6.59	39778419
22	*MGAT3*	1056	IGP11	rs5757678	3.40 x 10^{-13}	A, G (0.27)	0.03 (0.005)	0.05	7.37	39843409
22	*MGAT3*	1045	IGP11	rs5757680	1.63 x 10^{-13}	G, A (0.26)	0.04 (0.005)	0.05	7.48	39844793
22	*MGAT3*	1056	IGP15	rs1010169	1.51 x 10^{-11}	G, A (0.34)	0.1 (0.014)	0.04	6.82	39778167
22	*MGAT3*	1052	IGP15	rs137686	6.66 x 10^{-10}	G, C (0.25)	0.1 (0.016)	0.04	6.23	39739638
22	*MGAT3*	1056	IGP15	rs137690	6.07 x 10^{-10}	G, C (0.25)	0.1 (0.016)	0.04	6.25	39742825
22	*MGAT3*	1048	IGP15	rs137702	2.88 x 10^{-10}	G, A (0.24)	0.1 (0.016)	0.04	6.37	39750158

22	*MGAT3*	1056	IGP15	rs137707	3.66×10^{-10}	G, A (0.24)	0.1 (0.016)	0.04	6.33	39751401
22	*MGAT3*	1056	IGP15	rs2413589	5.36×10^{-10}	G, A (0.24)	0.1 (0.016)	0.04	6.27	39755689
22	*MGAT3*	1056	IGP15	rs4821887	3.66×10^{-10}	A, G (0.24)	0.1 (0.016)	0.04	6.33	39756985
22	*MGAT3*	1056	IGP15	rs5757650	3.60×10^{-11}	G, A (0.35)	0.09 (0.014)	0.04	6.69	39778419
22	*MGAT3*	1056	IGP15	rs5757678	3.98×10^{-15}	A, G (0.27)	0.12 (0.015)	0.06	7.97	39843409
22	*MGAT3*	1045	IGP15	rs5757680	1.04×10^{-15}	G, A (0.26)	0.12 (0.015)	0.06	8.15	39844793
22	*MGAT3*	1056	IGP15	rs9611156	9.50×10^{-10}	A, C (0.25)	0.1 (0.015)	0.03	6.17	39743170
22	*MGAT3*	1056	IGP19	rs5757678	2.69×10^{-10}	A, G (0.27)	0.1 (0.016)	0.04	6.38	39843409
22	*MGAT3*	1045	IGP19	rs5757680	1.38×10^{-10}	G, A (0.26)	0.1 (0.016)	0.04	6.48	39844793
22	*MGAT3*	1056	IGP23	rs5757678	6.16×10^{-11}	A, G (0.27)	-0.19 (0.028)	0.04	-6.61	39843409
22	*MGAT3*	1045	IGP23	rs5757680	5.04×10^{-11}	G, A (0.26)	-0.19 (0.028)	0.04	-6.64	39844793
22	*MGAT3*	1056	IGP24	rs5757678	1.00×10^{-11}	A, G (0.27)	0.12 (0.017)	0.04	6.88	39843409
22	*MGAT3*	1045	IGP24	rs5757680	7.65×10^{-12}	G, A (0.26)	0.12 (0.017)	0.04	6.92	39844793

The results are reported for the GRCh Build 37, and alleles are aligned to the TOP strand. The β coefficient is reported for the minor allele and expressed as the relative glycan abundance (%).

Chr, chromosome; N, sample size; MA, minor allele; OA, other allele; MAF, minor allele frequency; R^2, percentage of explained glycan variance; BP, position of a SNP on a chromosome in base-pairs; *ST6GAL1*, ST6 beta-galactoside alpha-2,6-sialyltransferase 1 gene; *MGAT3*, beta-1,4-mannosyl-glycoprotein 4-beta-*N*-acetylglucosaminyltransferase gene

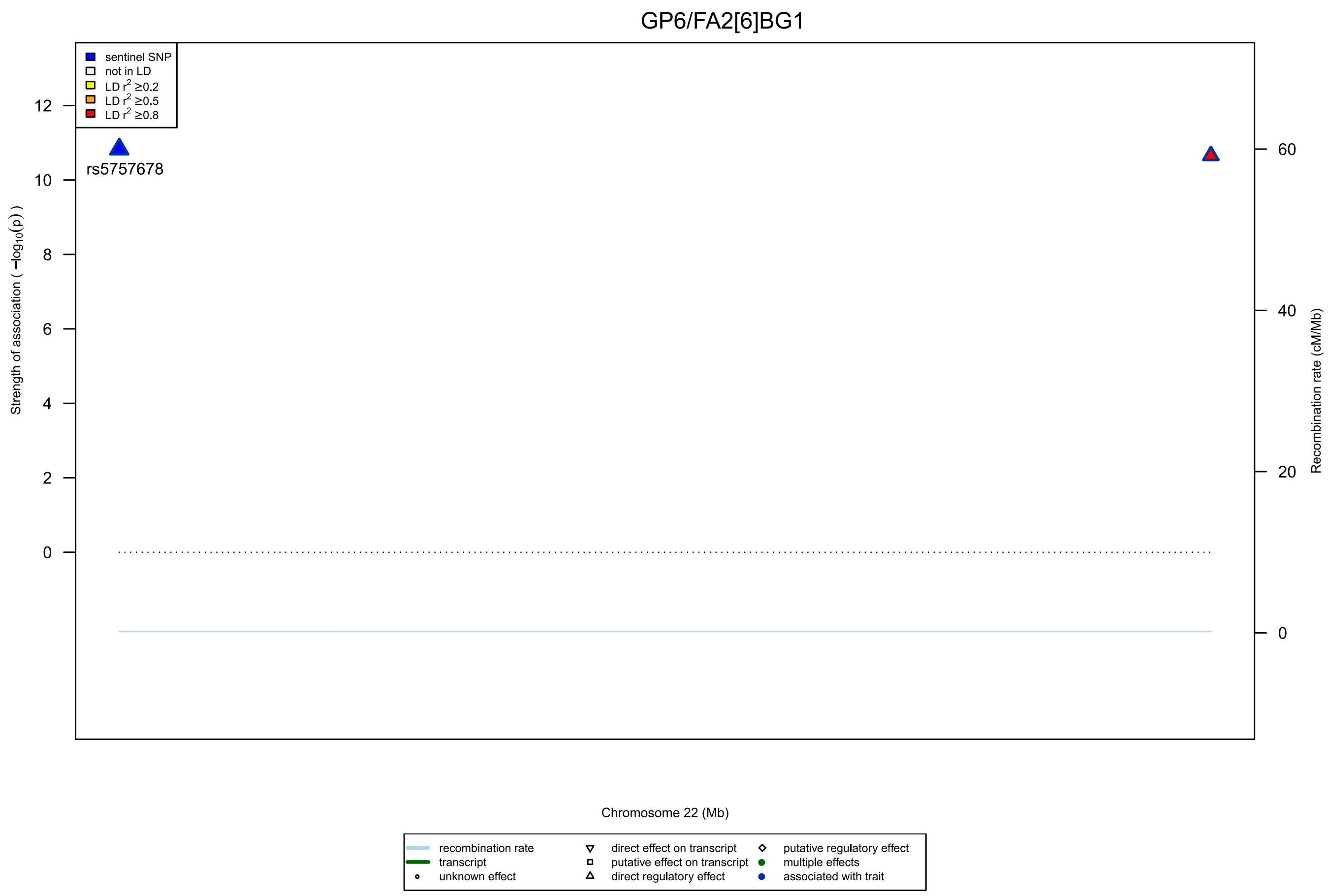

ESM Fig. 1 Regional association plot for GP6 glycan. Figure was created using SNiPA v3.4 from November 2020, GRCh37.p13, Ensembl version 87, 1000 genomes phase 3 v5, European (Arnold M, Raffler J, Pfeufer A, Suhre K, Kastenmüller G (2015) SNiPA: an interactive, genetic variant-centered annotation browser. Bioinforma 31(8):1334–1336). The plot shows the strength of association (-$\log_{10}$(P)), recombination rate, genes, and regulatory elements. The variants' symbols depict the functional annotations, the colors depict the pairwise LD correlations to the sentinel variant

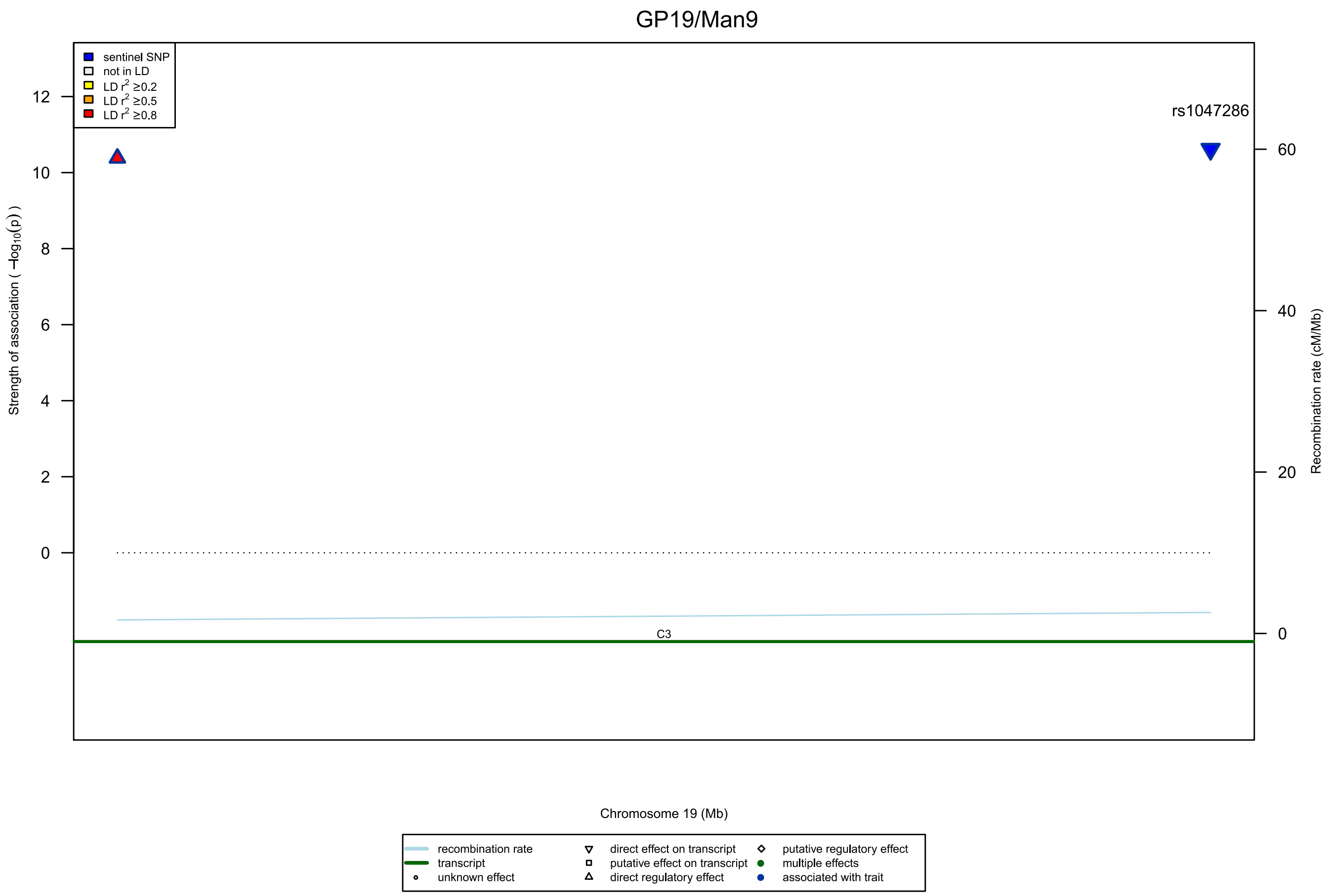

ESM Fig. 2 Regional association plot for GP19 glycan. Figure was created using SNiPA v3.4 from November 2020, GRCh37.p13, Ensembl version 87, 1000 genomes phase 3 v5, European (Arnold M, Raffler J, Pfeufer A, Suhre K, Kastenmüller G (2015) SNiPA: an interactive, genetic variant-centered annotation browser. Bioinforma 31(8):1334–1336). The plot shows the strength of association (-$\log_{10}$(P)), recombination rate, genes, and regulatory elements. The variants' symbols depict the functional annotations, the colors depict the pairwise LD correlations to the sentinel variant. *C3*, complement C3 gene

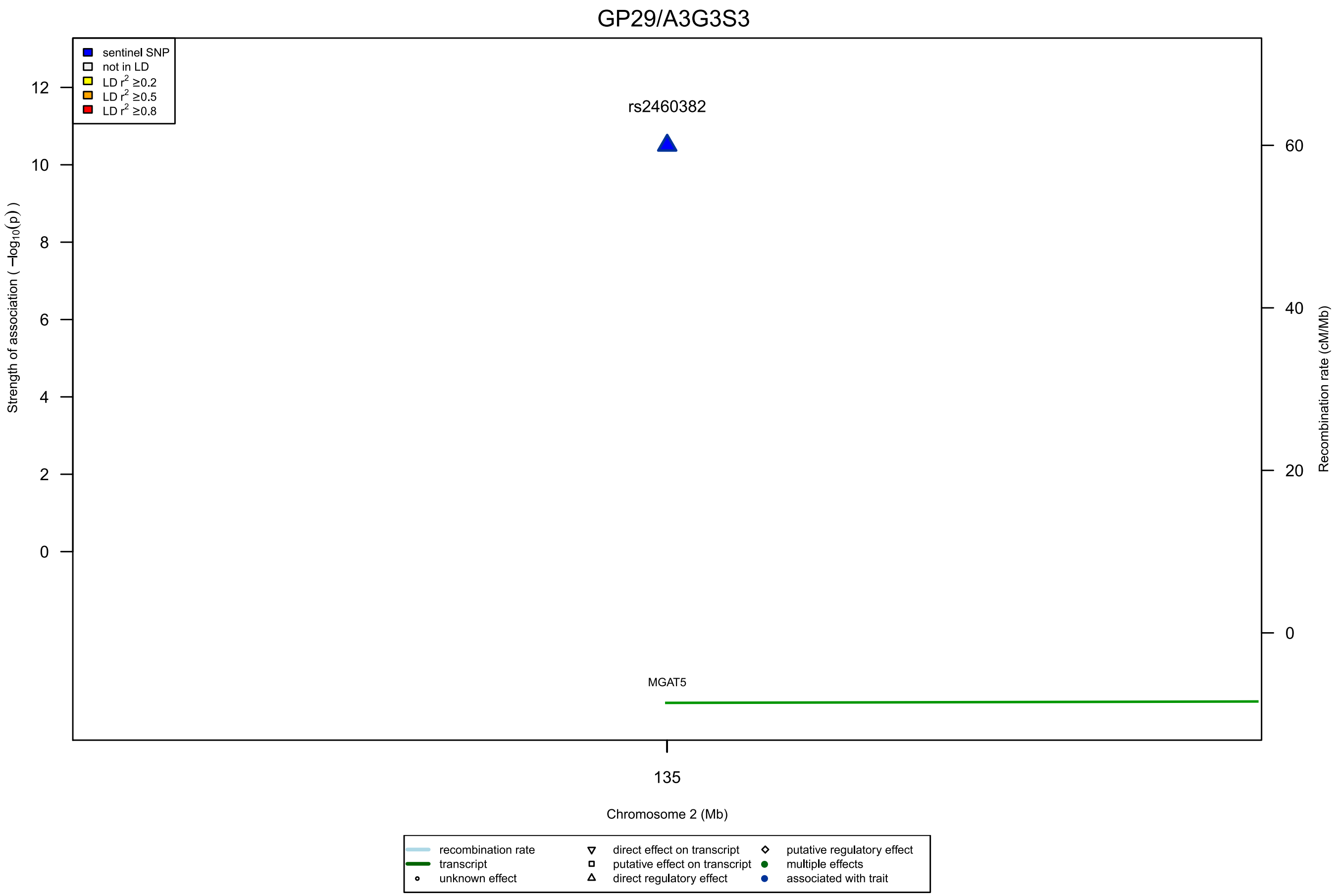

ESM Fig. 3 Regional association plot for GP29 glycan. Figure was created using SNiPA v3.4 from November 2020, GRCh37.p13, Ensembl version 87, 1000 genomes phase 3 v5, European (Arnold M, Raffler J, Pfeufer A, Suhre K, Kastenmüller G (2015) SNiPA: an interactive, genetic variant-centered annotation browser. Bioinforma 31(8):1334–1336). The plot shows the strength of association (-$\log_{10}$(P)), recombination rate, genes, and regulatory elements. The variants' symbols depict the functional annotations, the colors depict the pairwise LD correlations to the sentinel variant. *MGAT5*, alpha-1,6-mannosylglycoprotein 6-beta-*N*-acetylglucosaminyltransferase gene

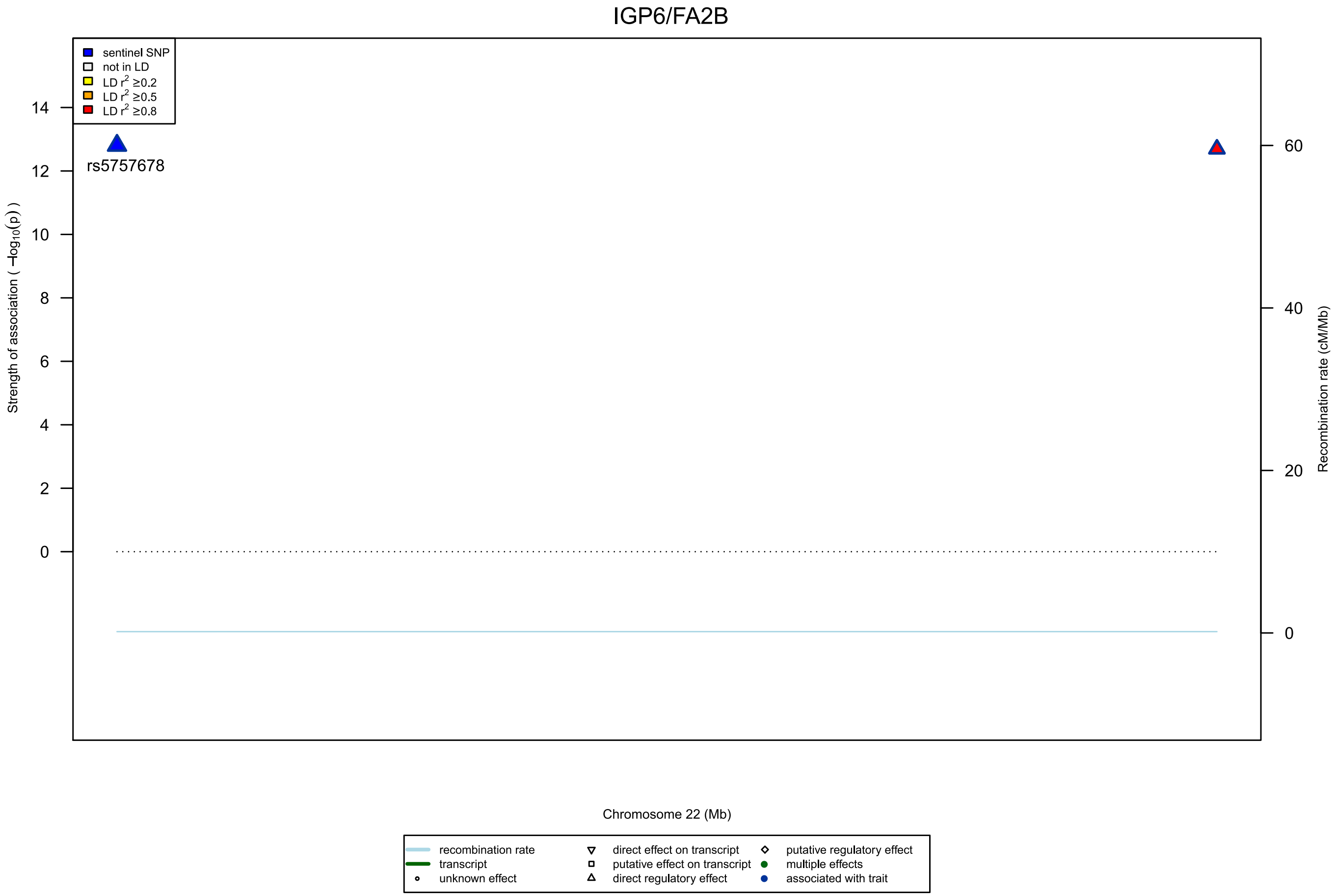

ESM Fig. 4 Regional association plot for IGP6 glycan. Figure was created using SNiPA v3.4 from November 2020, GRCh37.p13, Ensembl version 87, 1000 genomes phase 3 v5, European (Arnold M, Raffler J, Pfeufer A, Suhre K, Kastenmüller G (2015) SNiPA: an interactive, genetic variant-centered annotation browser. Bioinforma 31(8):1334–1336). The plot shows the strength of association ($-\log_{10}(P)$), recombination rate, genes, and regulatory elements. The variants' symbols depict the functional annotations, the colors depict the pairwise LD correlations to the sentinel variant

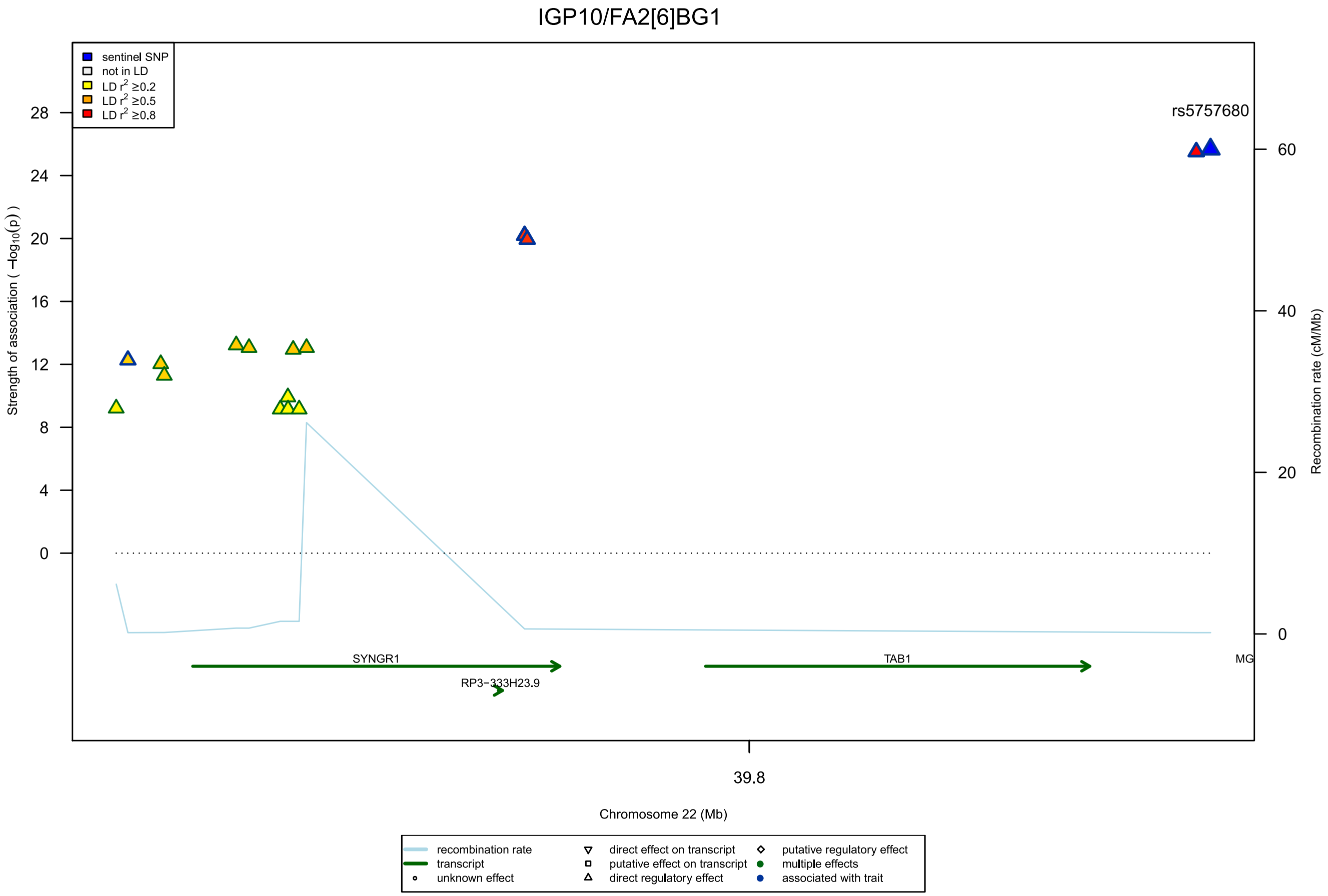

ESM Fig. 5 Regional association plot for IGP10 glycan. Figure was created using SNiPA v3.4 from November 2020, GRCh37.p13, Ensembl version 87, 1000 genomes phase 3 v5, European (Arnold M, Raffler J, Pfeufer A, Suhre K, Kastenmüller G (2015) SNiPA: an interactive, genetic variant-centered annotation browser. Bioinforma 31(8):1334–1336). The plot shows the strength of association (-$\log_{10}$(P)), recombination rate, genes, and regulatory elements. The variants' symbols depict the functional annotations, the colors depict the pairwise LD correlations to the sentinel variant. *SYNGR1*, synaptogyrin 1 gene; *TAB1*, TGF-beta activated kinase 1 (MAP3K7) binding protein 1 gene

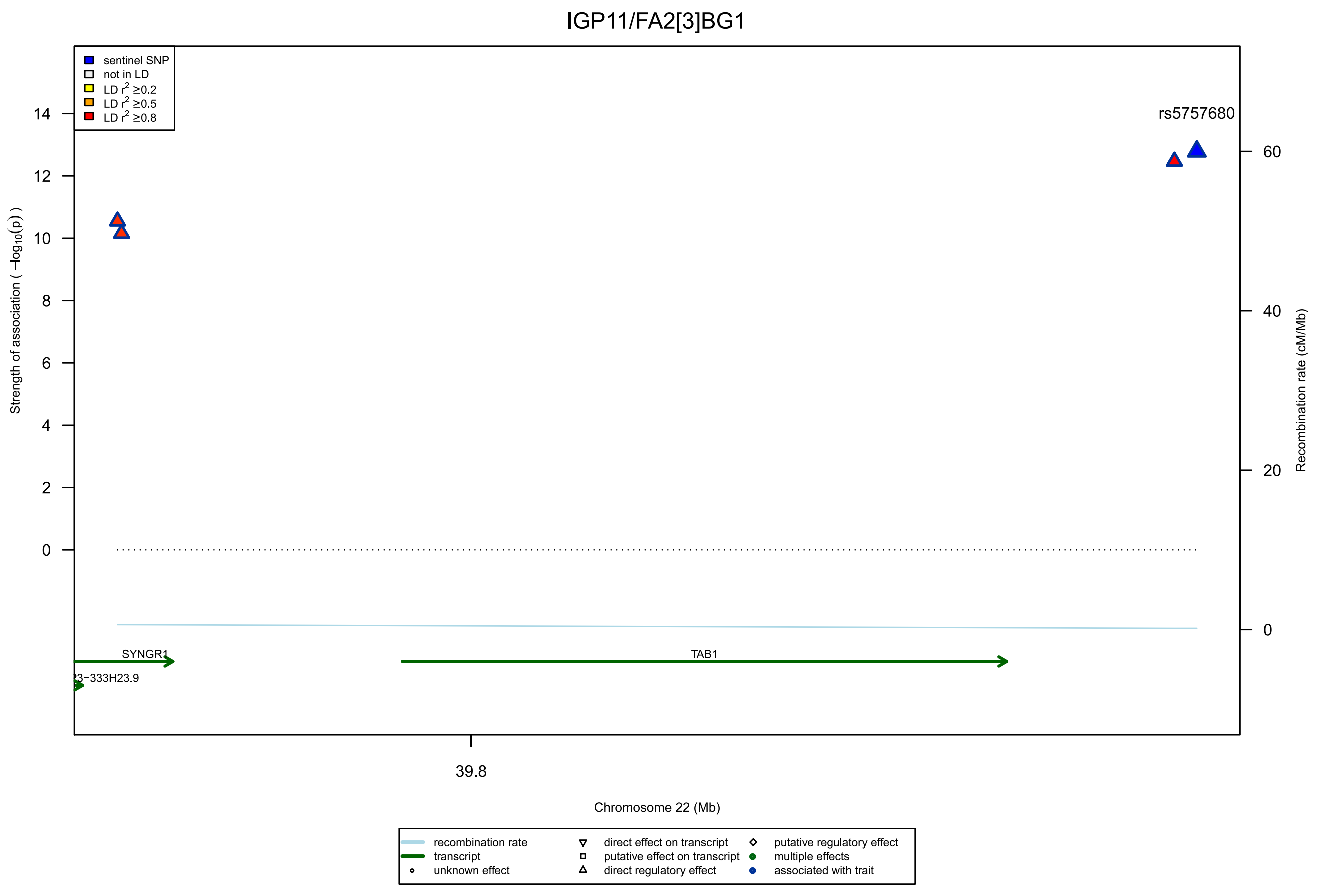

ESM Fig. 6 Regional association plot for IGP11 glycan. Figure was created using SNiPA v3.4 from November 2020, GRCh37.p13, Ensembl version 87, 1000 genomes phase 3 v5, European (Arnold M, Raffler J, Pfeufer A, Suhre K, Kastenmüller G (2015) SNiPA: an interactive, genetic variant-centered annotation browser. Bioinforma 31(8):1334–1336). The plot shows the strength of association (-$\log_{10}$(P)), recombination rate, genes, and regulatory elements. The variants' symbols depict the functional annotations, the colors depict the pairwise LD correlations to the sentinel variant. *SYNGR1*, synaptogyrin 1 gene; *TAB1*, TGF-beta activated kinase 1 (MAP3K7) binding protein 1 gene

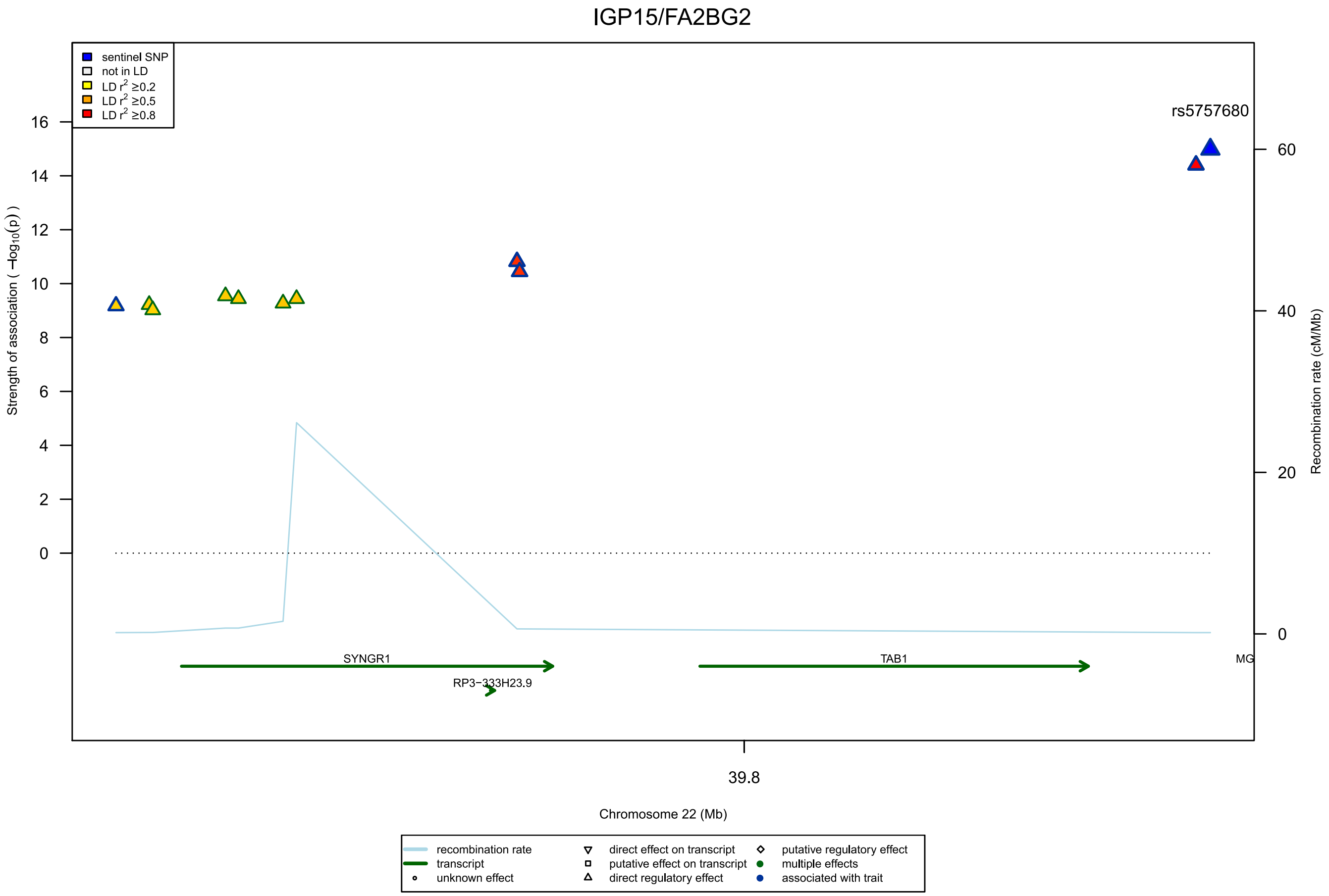

ESM Fig. 7 Regional association plot for IGP15 glycan. Figure was created using SNiPA v3.4 from November 2020, GRCh37.p13, Ensembl version 87, 1000 genomes phase 3 v5, European (Arnold M, Raffler J, Pfeufer A, Suhre K, Kastenmüller G (2015) SNiPA: an interactive, genetic variant-centered annotation browser. Bioinforma 31(8):1334–1336). The plot shows the strength of association (-$\log_{10}$(P)), recombination rate, genes, and regulatory elements. The variants' symbols depict the functional annotations, the colors depict the pairwise LD correlations to the sentinel variant. *SYNGR1*, synaptogyrin 1 gene; *TAB1*, TGF-beta activated kinase 1 (MAP3K7) binding protein 1 gene

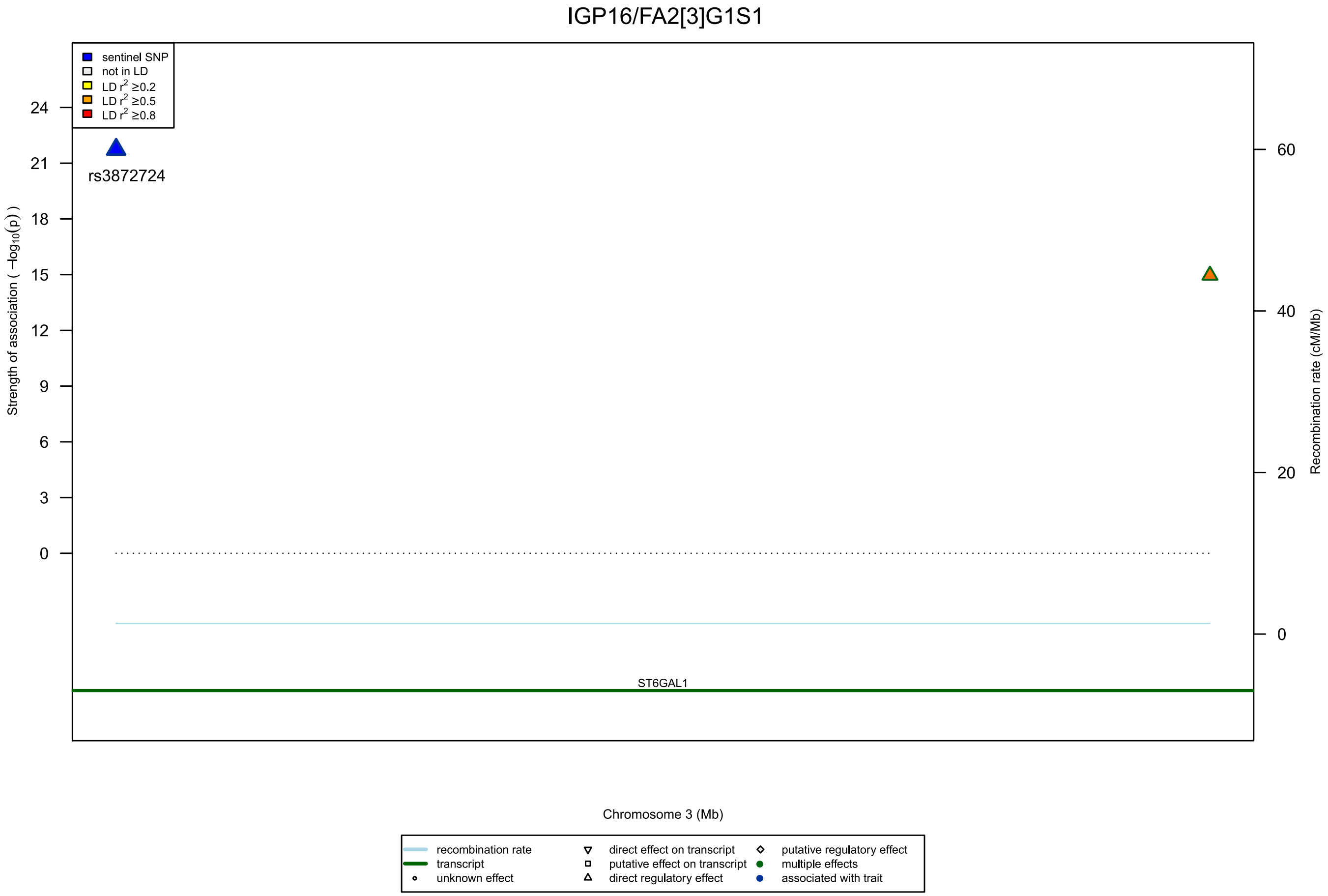

ESM Fig. 8 Regional association plot for IGP16 glycan. Figure was created using SNiPA v3.4 from November 2020, GRCh37.p13, Ensembl version 87, 1000 genomes phase 3 v5, European (Arnold M, Raffler J, Pfeufer A, Suhre K, Kastenmüller G (2015) SNiPA: an interactive, genetic variant-centered annotation browser. Bioinforma 31(8):1334–1336). The plot shows the strength of association (-$\log_{10}$(P)), recombination rate, genes, and regulatory elements. The variants' symbols depict the functional annotations, the colors depict the pairwise LD correlations to the sentinel variant. *ST6GAL1*, ST6 beta-galactoside alpha-2,6-sialyltransferase 1 gene

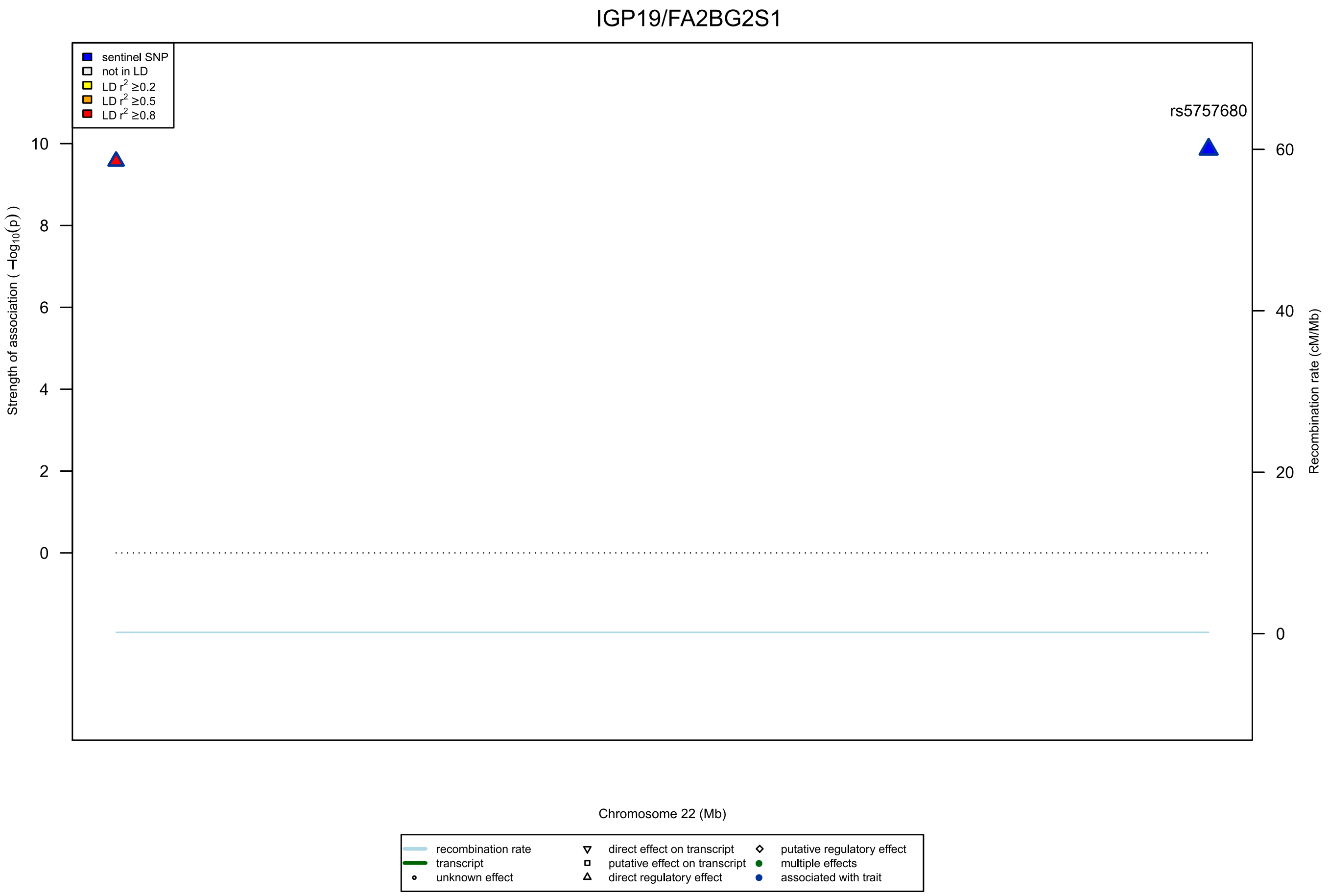

ESM Fig. 9 Regional association plot for IGP19 glycan. Figure was created using SNiPA v3.4 from November 2020, GRCh37.p13, Ensembl version 87, 1000 genomes phase 3 v5, European (Arnold M, Raffler J, Pfeufer A, Suhre K, Kastenmüller G (2015) SNiPA: an interactive, genetic variant-centered annotation browser. Bioinforma 31(8):1334–1336). The plot shows the strength of association ($-\log_{10}(P)$), recombination rate, genes, and regulatory elements. The variants' symbols depict the functional annotations, the colors depict the pairwise LD correlations to the sentinel variant

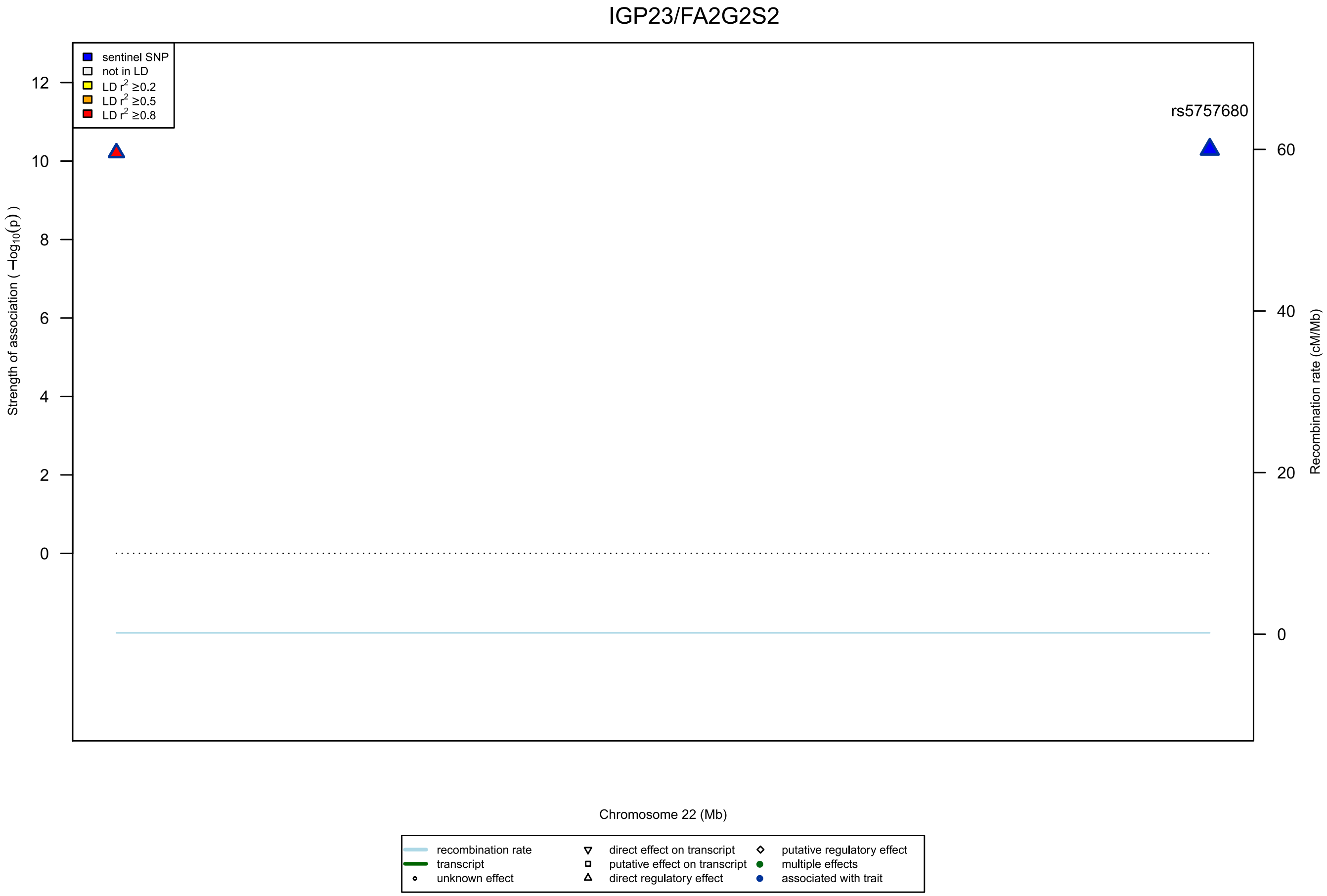

ESM Fig. 10 Regional association plot for IGP23 glycan. Figure was created using SNiPA v3.4 from November 2020, GRCh37.p13, Ensembl version 87, 1000 genomes phase 3 v5, European (Arnold M, Raffler J, Pfeufer A, Suhre K, Kastenmüller G (2015) SNiPA: an interactive, genetic variant-centered annotation browser. Bioinforma 31(8):1334–1336). The plot shows the strength of association (-$\log_{10}$(P)), recombination rate, genes, and regulatory elements. The variants' symbols depict the functional annotations, the colors depict the pairwise LD correlations to the sentinel variant

IGP24/FA2BG2S2

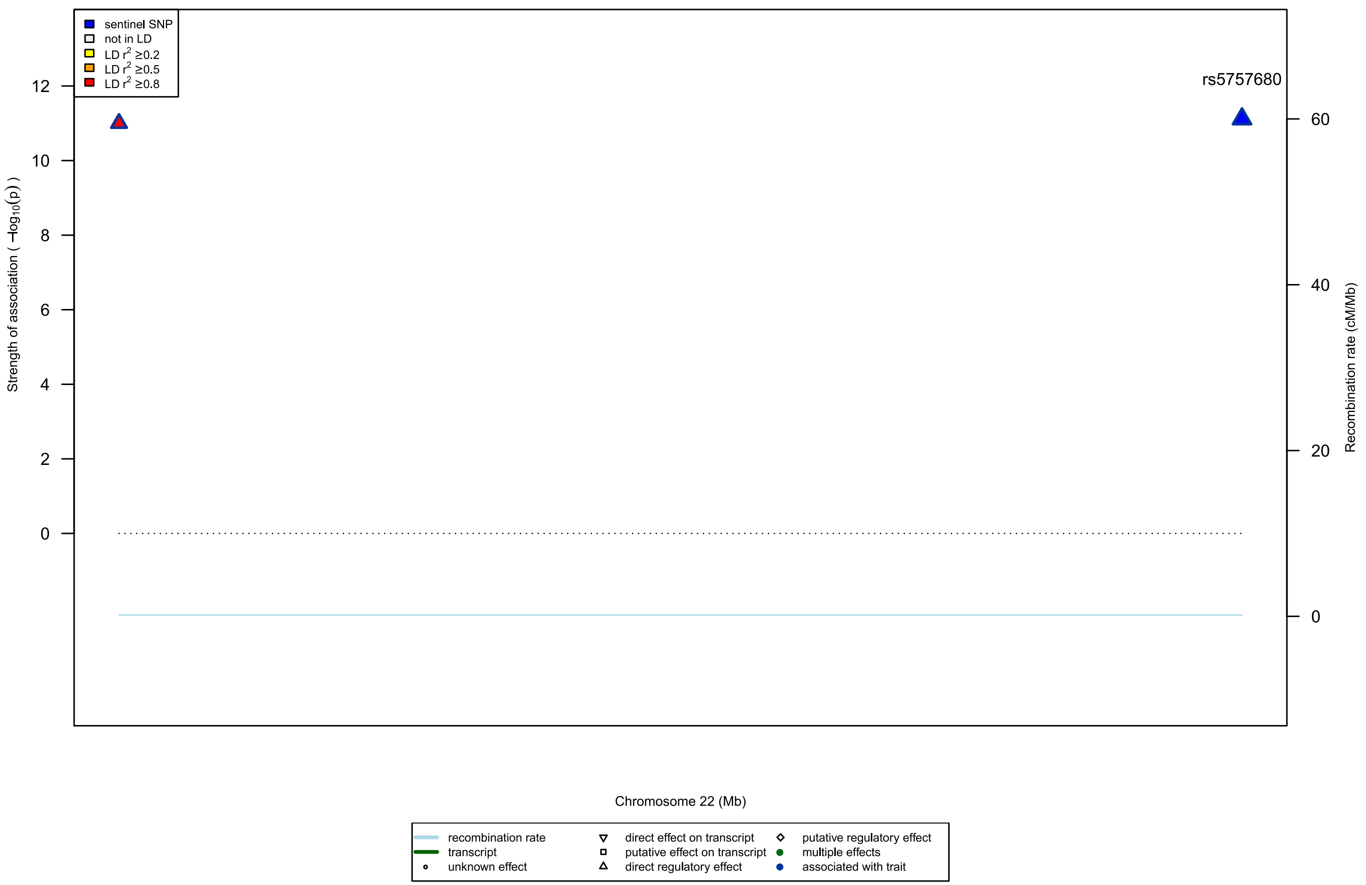

ESM Fig. 11 Regional association plot for IGP24 glycan. Figure was created using SNiPA v3.4 from November 2020, GRCh37.p13, Ensembl version 87, 1000 genomes phase 3 v5, European (Arnold M, Raffler J, Pfeufer A, Suhre K, Kastenmüller G (2015) SNiPA: an interactive, genetic variant-centered annotation browser. Bioinforma 31(8):1334–1336). The plot shows the strength of association (-log$_{10}$(P)), recombination rate, genes, and regulatory elements. The variants' symbols depict the functional annotations, the colors depict the pairwise LD correlations to the sentinel variant
